# Hyperparasitaemic human *Plasmodium knowlesi* infection with atypical morphology in peninsular Malaysia

**DOI:** 10.1186/1475-2875-12-88

**Published:** 2013-03-06

**Authors:** Wenn-Chyau Lee, Pek-Woon Chin, Yee-Ling Lau, Lit-Chein Chin, Mun-Yik Fong, Chee-Jiek Yap, Raymond Raj Supramaniam, Rohela Mahmud

**Affiliations:** 1 Tropical Infectious Diseases Research and Education Center (TIDREC), Department of Parasitology, Faculty of Medicine, University of Malaya, Kuala Lumpur, 50603, Malaysia; 2 Department of Medicine, Hospital Enche’ Besar Hajjah Khalsom, Kluang, Johor, Malaysia

**Keywords:** *Plasmodium knowlesi*, Mainland Southeast Asian strain, Malaria, Hyperparasitaemia, Amoeboid morphology, Treatment

## Abstract

*Plasmodium knowlesi* is a potentially life-threatening zoonotic malaria parasite due to its relatively short erythrocytic cycle. Microscopic identification of *P*. *knowlesi* is difficult, with “compacted parasite cytoplasm” being one of the important identifying keys. This report is about a case of hyperparasitaemic human *P*. *knowlesi* infection (27% parasitaemia) with atypical amoeboid morphology. A peninsular Malaysian was admitted to the hospital with malaria. He suffered anaemia and acute kidney function impairment. Microscopic examination, assisted by nested PCR and sequencing confirmed as *P*. *knowlesi* infection. With anti-malarial treatment and several medical interventions, patient survived and recovered. One-month medical follow-up was performed after recovery and no recrudescence was noted. This case report highlights the extreme hyperparasitaemic setting, the atypical morphology of *P*. *knowlesi* in the patient’s erythrocytes, as well as the medical interventions involved in this successfully treated case.

## Background

Malaria is caused by four species of human malaria parasites, namely *Plasmodium falciparum*, *Plasmodium vivax*, *Plasmodium malariae* and *Plasmodium ovale*. Recently, *P*. *knowlesi*, a species naturally found in long-tailed macaque (*Macaca fascicularis*) and pig-tailed macaque (*Macaca nemestrina*) [1,2], has been proven to be capable of causing infection in humans [3,4]. *P*. *knowlesi* infection is prevalent in Southeast Asia. Imported cases have been reported in European countries due to eco-tourism programmes to the forested areas of this region [5-7].

Owing to its 24-hour erythrocytic stage development [8,9], *P*. *knowlesi* has raised concern in the medical community regarding its high potential to replicate quickly, which may result in massive erythrocyte destruction in patients. Knowlesi malaria shows a fatality rate that is comparable, if not higher than that of falciparum malaria [10,11]. The severity of knowlesi malaria is significantly associated with high parasitaemia [10]. Nevertheless, knowlesi malaria cases with hyperparasitemic setting were not frequently found except in Malaysian Borneo [5,6,10-16]. Recently, a case of *P*. *knowlesi* reinfection in peninsular Malaysia was reported, with a parasitaemia of 2.0% during the first infection and 2.5% during the second infection [17]. *Plasmodium knowlesi* infection with extreme hyperparasitaemia, higher than that has never been reported in peninsular Malaysia. Microscopic identification of *P*. *knowlesi* is difficult for many laboratory technicians due to the overlapping morphology with that of other human malaria parasites, such as *P*. *falciparum* and *P*. *malariae*[3,10,12,13,18]. Here, the first hyperparasitaemia with atypical amoeboid morphology in a knowlesi malaria case from peninsular Malaysia is reported. Diagnosis was confirmed using microscopic examination of blood smear, polymerase chain reaction (PCR) and sequencing. The treatment strategy applied for this case, and the sequence of diagnostic steps in identifying *P*. *knowlesi* infection are described.

## Case presentation

A 56-year-old Chinese man was admitted to Hospital Enche’ Besar Hajjah Khalsom, Kluang, Johor with a history of five days of high grade fever, including two days of yellowish discoloration of the skin and extreme tiredness. He worked as a sawmill supervisor at Kluang, Johor. He had no travel history to other countries or deep jungles in Malaysia. However, he often visited a recreational park at the foothill of Gunung Lambak, Kluang, Johor.

Upon admission, he was alert, conscious but appeared lethargic. There was neither neck stiffness nor papilloedema on fundus examination. He was febrile with temperature of 38°C. He had mild pallor, deep jaundice but no petechiae. He was haemodynamically stable, with well perfused peripherals, no signs of tachypnea but the Pulse oximetry (SpO_2_) monitoring was 72–85% despite good oxygenation on arterial blood gases analysis for about 24 hours after admission. Physical examination of cardiovascular and respiratory systems were normal. He had hepatomegaly, but no splenomegaly.

The initial haematological investigations revealed mild anaemia (Hb level 10.0 g/dL), thrombocytopaenia (48,000 platelets/μl) with normal total white count and haematocrit. Dengue tests for NS1 Ag, IgM and IgG were negative. Serology tests for Hepatitis B surface antigen (HepBsAg), Anti-HCV and HIV tests were negative. Renal function was abnormal as evidenced by high urea and creatinine levels. Liver enzymes were elevated with hyperbilirubinemia of mixed picture. Lactate dehydrogenase (LDH) level was elevated but reticulocyte count was normal and Coombs test was negative. The initial chest radiography revealed normal lung field and no pulmonary congestion.

**Figure 1:**
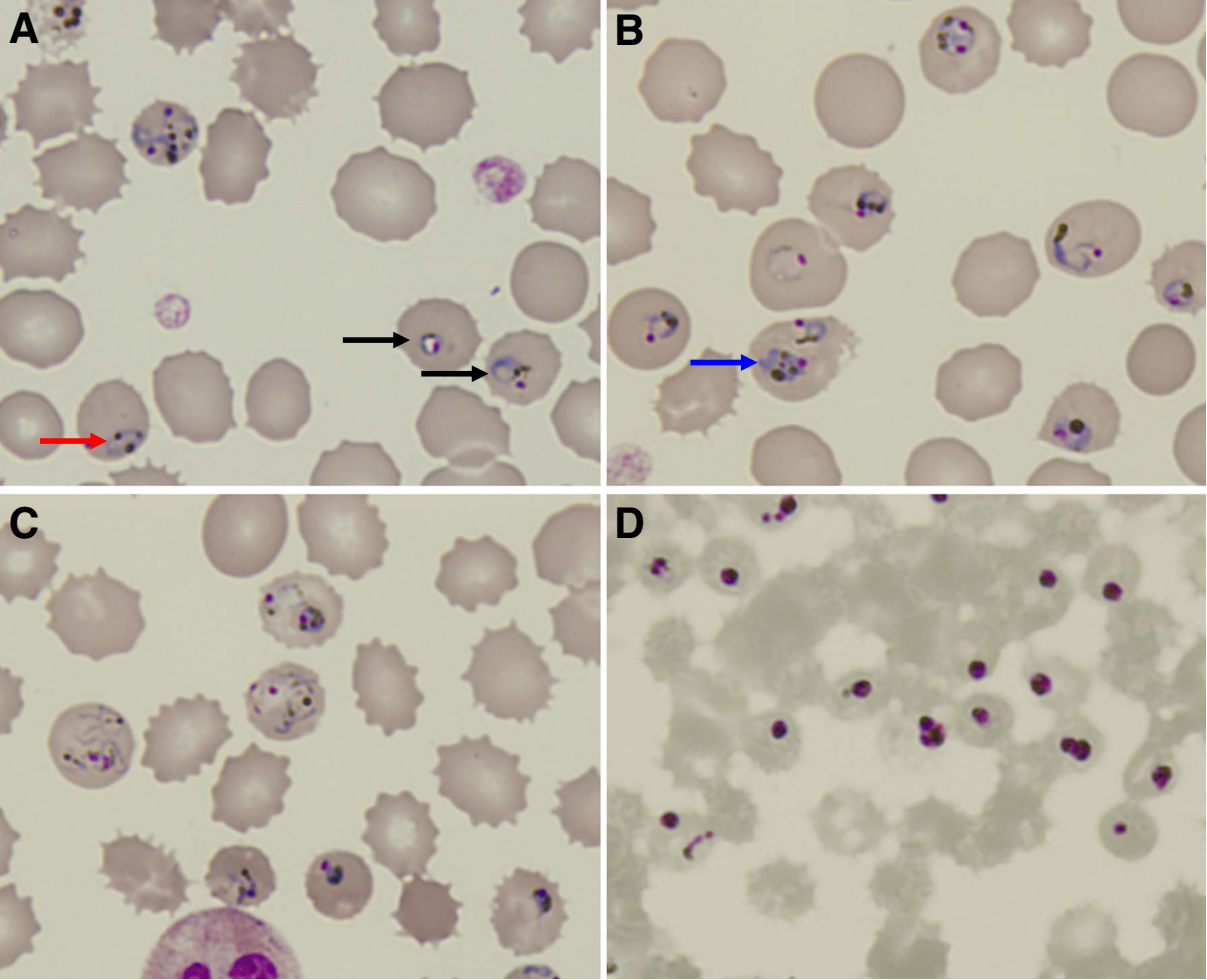
**Blood smear before treatment (A-C) and 1 day post-treatment (D).** Arrow in red shows parasite with distantly parted chromatin dots, whereas arrow in black shows parasite with chromatin dot located within the “ring” form (**A**). Multiple-infection was found (**B**). Compacted “band” form trophozoite can be seen here (pointed by blue arrow). *P*. *knowlesi* in highly amoeboid forms are shown in (**C**). The characteristic golden-brownish pigments can be seen within the parasites.

Rapid diagnosis with BinaxNOW® malaria diagnostic kit showed positive T2 band, indicating infection caused by non-*P*. *falciparum* malaria parasites. Thick and thin Giemsa-stained blood smears examination unravelled abundant parasitized erythrocytes. The developmental stage of the parasites was not synchronized. Many parasites showed highly amoeboid morphology. Presence of numerous golden brown pigments with no enlargement of infected erythrocytes was indicative of *P*. *knowlesi* infection (Figure 1A-C). Based on the thin blood smear counting, the parasitemia level was 27%. Such overwhelmingly high parasitemia may be related to the patient’s 5 days of high grade fever prior to hospital admission. For a species with quotidian erythrocytic cycle, 5 days without proper medical intervention would be adequate for *P*. *knowlesi* to replicate and increase its population within the host to such a high value.

PCR was conducted to confirm the species of malaria parasite. DNA was extracted and purified from 100 μl of patient’s whole blood using the DNeasy Blood & Tissue Kit (QIAGEN, Valencia, CA). The nested PCR was performed with primers developed previously from 18S ribosomal RNA (18S rRNA) gene [3]. The nest 1 reaction mixture of 25 μl contained 4 μl of DNA template, 5 pmol of genus-specific primers (rPLU1: 5’-TCA AAG ATT AAG CCA TGC AAG TGA-3’and rPLU5: 5’-CCT GTT GTT GCC TTA AAC TCC-3’), 1X PCR Buffer (35 mM Tris-HCl, pH 9.0, 3.5 mM MgCl_2_, 25 mM KCl, 0.01% gelatin), 0.25 M dNTP and 1 u *Taq* polymerase and 15.3 μl of nuclease free water. The PCR conditions were as follows: (1) Initial denaturation at 94°C for 4 minutes, (2) 35 cycles of denaturation at 94°C for 30 seconds, annealing at 55°C for 1 minute and extension at 72°C for 1 minute, (3) final extension at 72°C for 10 minutes and a hold temperature of 4°C. The nest 2 amplification mixture contained 4 μl of the nest 1 product and same amounts of buffer, dNTP, *Taq* polymerase and nuclease free water as in nest 1. The primer sets used in nest 2 PCR were as follow: FAL1: 5’-TTA AAC TGG TTT GGG AAA ACC AAA TAT ATT-3’ and FAL2: 5’-ACA CAA TGA ACT CAA TCA TGA CTA CCC GTC-3’ for *P*. *falciparum*; VIV1: 5’-CGC TTC TAG CTT AAT CCA CAT AAC TGA TAC-3’ and V1V2: 5’-ACT TCC AAG CCG AAG CAA AGA AAG TCC TTA-3’ for *P*. *vivax*; MAL1: 5’-ATA ACA TAG TTG TAC GTT AAG AAT AAC CGC-3’ and MAL2: 5’-AAA ATT CCC ATG CAT AAA AAA TTA TAC AAA- 3’ for *P*. *malariae*; OVAL1: 5’-ATC TCT TTT GCT ATC TTT TTT TAG TAT TGG AGA- 3’ and OVAL2: 5’-GGA AAA GGA CAC ATT AAT TGT ATC CTA GTG-3’ for *P*. *ovale*; Pmk8: 5’-GTT AGC GAG AGC CAC AAA AAA GCG AAT-3’ and Pmkr9: 5’-ACT CAA AGT AAC AAA ATC TTC CGT A-3’ for *P*. *knowlesi*. The nest 2 amplification conditions were similar to that of nest 1 except that the annealing temperature of 58°C was used for the species-specific primers. The PCR products were purified with QIAquick Gel Extraction Kit (QIAGEN, Valencia, CA) and cloned into pGEM®-T Vector Systems (Promega, Wisconsin, USA), before sending to DNA sequencing service provider. Ethical approval for this study was obtained from the Medical Ethics Committee of University Malaya Medical Centre (Ref No. 817.18).

**Figure 2:**
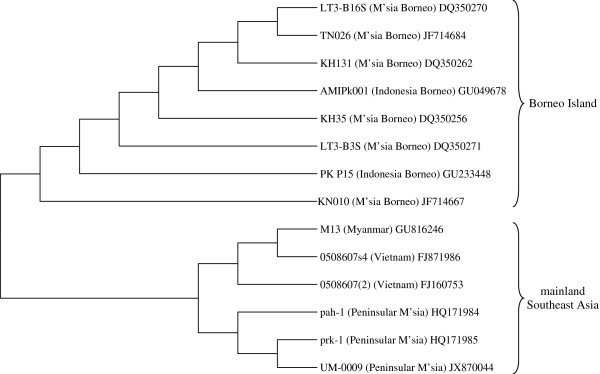
**Phylogenetic tree based on 18S rRNA gene sequences of *Plasmodium knowlesi *isolates from mainland Southeast Asia and Borneo Island.** Isolate UM-0009 in our case report is placed in the mainland Southeast Asia cluster. The tree is constructed using the Neighbor-Joining method (bootstrap = 1000) which is available in MEGA4 [16]. GenBank Accession number is given after each isolate’s name.

Nested PCR showed that the blood sample was positive for *P*. *knowlesi* infection. Distinct bands of 153 bp was observed in the agarose gel electrophoresis for the nested PCR. Sequence analysis with Basic Local Alignment Search Tool [19] confirmed that the sample is positive with *P*. *knowlesi* infection. A phylogenetic tree was constructed based on the 18S rRNA gene sequences of *P*. *knowlesi* isolates from mainland Southeast Asia and Borneo Island using the neighbour-joining method (bootstrap = 1000) [20]. Isolate UM-0009 (accession number JX870044) in this report is placed in the mainland Southeast Asia cluster (Figure 2). This shows that *P*. *knowlesi* isolates from the mainland Southeast Asia are capable of causing infections with extremely high parasitemia as well.

The patient was treated right after admission with four doses of intravenous (IV) artesunate at 2.4 mg/kg at 0, 12, 24 and 48 hours in combination with oral doxycycline 100 mg BD for 1 week duration. Packed cell transfusions were given to the patient due to development of anaemia. Unfortunately, the patient went into oliguric phase of acute kidney injury. Under ultrasound examination, both kidneys showed increased parenchymal echogenicity, suggestive of bilateral parenchymal disease. The right kidney measured 9.6 cm with cortical thickness of 1.7 cm. The left kidney was 9.5 cm with cortical thickness of 2.0 cm. Calculus and hydronephrosis were not noted in both kidneys. Patient’s prostate glands showed no enlargement. He needed 7 sessions of hemodialysis. Eventually, he recovered almost completely from acute kidney injury. His renal function returned to almost normal after one month of illness. On the second day after administration of the anti-malarial therapy, repeated blood smear was done. The smear revealed dead and unhealthy parasites, characterized by “diffused” nuclei and shrinkage of parasite (Figure 1D). The parasites count declined by four to five-fold for every 24 hours after treatment. No malaria parasite was noted in the peripheral blood film from day 5 onwards. There was no recrudescence after the illness based on one month of clinical follow-up (Table 1).

**Table 1:** One-month-clinical follow up investigations performed on the patient of this case

**Day**	**1**	**2**	**3**	**4**	**5**	**21**	**33**
**Hb (g/dL)**	10.00	10.90	6.71	10.80	10.00	8.45	9.64
{13.80–17.20}							
**TWBC (× 10**^**3 **^**/μl)**	10.50	12.50	13.20	14.40	19.00	5.44	8.65
{3.80–10.80}							
**Platelet (× 10**^**3**^**/μl)**	48.00	33.40	81.10	124.00	96.50	396.00	498.00
{150.00–450.00}							
**Urea (mg/dL)**	24.60	32.30	34.30	23.00	24.00	26.40	11.90
{7.00–30.00}							
**Potassium (mmol/L)**	4.45	4.54	4.19	4.40	3.68	3.24	3.68
{3.50–5.00}							
**Sodium (mmol/L)**	120.00	122.00	119.00	131.00	130.00	130.00	134.00
{135.00–145.00}							
**Creatinine (μmol/L)**	371.00	486.00	494.00	471.00	557.00	739.00	179.00
{≤106}							
**TB (μmol/L)**	155.10	80.00	85.00	31.10	28.80	N/A	14.90
{<26}							
**ALP (U/L)**	203.00	178.00	155.00	147.00	147.00	N/A	107.00
{40.00–120.00}							
**Albumin (g/L)**	31.00	27.00	23.00	24.00	22.00	N/A	39.00
{3.50–5.00}							
**AST (U/L)**	212.00	174.00	295.00	177.00	89.00	N/A	34.00
{0–37.00}							
**ALT (U/L)**	89.00	82.00	104.00	102.00	75.00	N/A	36.00
{0–35.00}							
**pH**	7.50	7.47	N/A	N/A	N/A	N/A	N/A
{7.34–7.44}							
**PaCO**_**2 **_**(mmHg)**	26.00	21.00	N/A	N/A	N/A	N/A	N/A
{35.00–45.00}							
**PaO**_**2 **_**(mmHg)**	168.00	109.00	N/A	N/A	N/A	N/A	N/A
{75.00–100.00}							
**HCO**_**3 **_**(mmol/L)**	22.80	18.50	N/A	N/A	N/A	N/A	N/A
{22.00–26.00}							
**LDH (U/L)**	1329.00	N/A	1613.00	N/A	N/A	N/A	N/A
{105.00–230.00}							
**Parasitemia (%)**	27.00	4.50	0.80	0.15	0	0	0

Values in {} indicate the normal range of the respective subjects tested, corresponding to the age and gender of our patient. Hb = Haemoglobin; TWBC = Total White Blood Cell Count; TB = Total Bilirubin; ALP = Alkaline Phosphatase; AST = Aspartate Aminotransferase; ALT = Alanine Aminotransferase; PaCO_2_ = Arterial Carbon Dioxide Partial Pressure; PaO_2_ = Arterial Oxygen Partial Pressure; LDH = Lactate Dehydrogenase; N/A = Not Available.

For malaria diagnosis, light microscopic examination of thin blood smear is still the gold standard. The morphology of *P*. *knowlesi* overlaps with that of *P*. *falciparum* and *P*. *malariae* at different erythrocytic stages [1,2,13,18,21]. Therefore, microscopic identification of *P*. *knowlesi* is extremely difficult even with experienced laboratory personnel. Nevertheless, this method still contributes significantly to the species identification in malaria infection. With complete patient’s travel history, rapid diagnostic test, peripheral blood platelet characterization and parasiteamia counting, microscopic examination still serves as part of the first-line method to verify the aetiological agent of the infection. For this case report, the identification keys for *P*. *knowlesi* were obtained from previous studies conducted on monkeys and humans [1,2,9,13,21]. Unlike the observation in other studies, the blood smear in this case shows abundant parasites in highly amoeboid form (Figure 1C). Presence of malaria parasites with highly amoeboid morphology should not be straight forwardly determined as *P*. *vivax*, which is often accompanied with less fatal outcomes.

For this case report, the anti-malarial regime of IV artesunate and doxycycline worked efficiently in killing most of the parasites within one day after the course of treatment. Therefore, this formulation can serve as an alternative regime for treating knowlesi malaria besides the treatment formulation mentioned in previous studies [9,12,14], especially for patients with Glucose-6-phosphate Dehydrogenase (G6PD) deficiency, who cannot use quinine. Due to its fast quotidian erythrocytic cycle, *P*. *knowlesi* infection carries high risk of acute haemolytic anaemia that is potentially fatal. Therefore, patients infected with *P*. *knowlesi* should be treated immediately and have their haematological profile monitored closely from time to time. Meanwhile, blood transfusion may be a crucial supplementary strategy in treating patients with hyperparasitemia. Blood transfusion assists by compensating the destruction of erythrocytes upon rupture of schizonts. This is especially important for patients with compromised bone marrow erythropoietic activity. Indeed, such medical intervention was mentioned previously in treatment of complicated knowlesi malaria [12]. The causes of acute kidney injury in this patient may be due to the combination of factors, which include hypovolemia secondary to dehydration, hyperparasitemia, and haemoglobinaemia arising from haemolysed infected erythrocytes. During this period of time, hemodialysis was vital for patient’s survival as his renal function was impaired. Since renal involvement is not rare in pathogenesis of *P*. *knowlesi* infection [10-12], it is important to note that patient care and treatment for malaria cases should not be focused solely on clearing the parasites, and should not stop right after the parasites are cleared completely from the blood stream. This is especially crucial for cases with hyperparasitemia.

As mentioned by previous studies, anaemia, thrombocytopaenia and abnormal renal function were noted in our patient [9-12]. Besides, the deep jaundice noticed on this patient upon admission is the result of large-scale haemolysis that leads to overproduction of bilirubin. Another finding from this case that is worth mentioning is the patient’s “anaemia accompanied with normal reticulocyte reading” upon admission. In general, the erythropoietic activity in the bone marrow will be up-regulated when large amount of erythrocytes are cleared from the blood stream as a compensatory mechanism. Consequently, the peripheral reticulocyte reading will increase. Therefore, reticulocyte count serves as an indicator of erythropoietic activity [22,23]. Normal reticulocyte reading in an anaemic setting suggests suppression of erythropoiesis. Unlike *P*. *falciparum*[24,25], the suppression of erythropoietic activity in human *P*. *knowlesi* infection has not been investigated thoroughly as to date. Therefore, the impact of *P*. *knowlesi* infection on the integrity of erythropoiesis deserves more attention in knowlesi malaria research.

Previously, cases reported from peninsular Malaysia and other places of mainland Southeast Asian region were mostly accompanied with low parasitaemia [5, 15, unpublished data]. This case report shows that *P*. *knowlesi* isolates from the mainland Southeast Asian regions are capable of causing hyperparasitemic infections. Nevertheless, with prompt and appropriate therapeutic intervention, the patient has a higher chance of recovery. With systematic and detailed study on thin smear using light microscope, supplemented with physical examination, preliminary identification of *P*. *knowlesi* is still possible, albeit with some difficulty.

## Conclusions

A successfully treated hyperparasitemic knowlesi malaria case with atypical amoeboid morphology from peninsular Malaysia is described. In view of the challenges in diagnosis, healthcare workers should be aware of such atypical amoeboid morphology in knowlesi malaria samples. Besides, combination of artesunate and doxycyline can be an alternative treatment regime for knowlesi malaria cases. The renal function of knowlesi malaria patients should be monitored closely throughout the course of treatment, even after the parasites are successfully cleared from the blood stream.

## Consent

Consent was granted by patient for the publication of this case report.

## Competing interests

The authors declare that they have no competing interests.

## Authors’ contributions

WCL, LCC, YLL, MYF and RM carried out laboratory works and analysed the data. PWC CJY and RRS collected blood samples, conducted clinical diagnoses and treatment intervention. WCL and YLL participated in the data analyses and helped to draft the manuscript. All authors read and approved the final manuscript.
